# Antioxidants from Plants Protect against Skin Photoaging

**DOI:** 10.1155/2018/1454936

**Published:** 2018-08-02

**Authors:** Ganna Petruk, Rita Del Giudice, Maria Manuela Rigano, Daria Maria Monti

**Affiliations:** ^1^Department of Chemical Sciences, University of Naples Federico II, Complesso Universitario Monte Sant'Angelo, via Cinthia 4, 80126 Naples, Italy; ^2^Department of Experimental Medical Science, Lund University, 221 84 Lund, Sweden; ^3^Department of Agricultural Sciences, University of Naples Federico II, via Università 100, 80055 Portici, Naples, Italy; ^4^Istituto Nazionale di Biostrutture e Biosistemi (INBB), Rome, Italy

## Abstract

Exposure to UV light triggers the rapid generation and accumulation of reactive oxygen species (ROS) in skin cells, with consequent increase in oxidative stress and thus in photoaging. Exogenous supplementation with dietary antioxidants and/or skin pretreatment with antioxidant-based lotions before sun exposure might be a winning strategy against age-related skin pathologies. In this context, plants produce many secondary metabolites to protect themselves from UV radiations and these compounds can also protect the skin from photoaging. Phenolic compounds, ascorbic acid and carotenoids, derived from different plant species, are able to protect the skin by preventing UV penetration, reducing inflammation and oxidative stress, and influencing several survival signalling pathways. In this review, we focus our attention on the double role of oxidants in cell metabolism and on environmental and xenobiotic agents involved in skin photoaging. Moreover, we discuss the protective role of dietary antioxidants from fruits and vegetables and report their antiaging properties related to the reduction of oxidative stress pathways.

## 1. Introduction

Reactive oxygen species (ROS) are normally produced in cell metabolism, but, when the balance between free radicals and antioxidants favours the former, they can also take part in a pathological process known as oxidative stress. Oxidative stress may result in cell damage, thus leading to the development of many types of diseases, as well as aging [1]. With aging, a decreased performance of cell endogenous antioxidant system occurs; thus, elderly people are more susceptible to oxidative stress [2, 3]. Several secondary plant metabolites are endowed with antioxidant activity and have been studied to prevent, retard, and control the development of age-related pathologies [4]. The skin is considered the largest organ with a protective role against external noxious sources, such as UV radiations. In particular, exposure to UV light triggers the rapid generation and accumulation of ROS in skin cells, which may result in photoaging. In this review, we focus our attention on the role of oxidants in their physiological context and in pathological conditions, with a special attention on skin photoaging. Then, the protective role of antioxidants from fruits and vegetables is discussed. Their antiaging properties, related to the activity of intracellular oxidative stress pathways, are reported.

## 2. Physiological Role of Oxidants

All life processes are governed by redox signalling; thus, the maintenance of a physiological level of oxidants is mandatory for proper cellular functioning. This can be obtained by switching on/off some regulation pathways or programmed cell death. Oxidants are responsible for a well-known process, senescence, as they are involved in telomere shortening. Different authors demonstrated that cells grown in the presence of strong oxidative environments have a shorter life span compared with cells grown in low oxygen tension [5–7]. Indeed, oxygen is one of the most abundant oxidants. This chemical element is necessary for all aerobic organisms and acts as terminal oxidant in the mitochondrial respiratory chain, which is the main source of energy for the cell [8]. In eukaryotic cells, oxygen can be partially reduced by several enzymatic and nonenzymatic reactions, thus inducing the production of reactive intermediates, such as superoxide radical (O_2_^•−^), peroxyl (ROO^•^), alkoxyl (RO^•^), and hydroxyl (HO^•^), better known as reactive oxygen species (ROS). All these molecules need to be stabilized by reacting with other molecules, such as nitric oxide (NO^•^), and forming reactive nitrogen species (RNS). This constitutes the basis for the formation of a multitude of additional oxidative signalling elements, including the highly reactive and potentially damaging peroxynitrite (ONOO^−^) [9, 10]. Both ROS and RNS may target cysteine thiols, leading to oxidative modifications and to the formation of reactive sulphur species (RSS) [11].

Despite this, a small, nontoxic increase in ROS levels plays a key role in the prevention of the insurgence of different diseases by assisting the immune system, mediating cell signalling, and playing an essential role in apoptosis [12]. Indeed, ROS can alter the mitochondrial membrane potential and induce the release of cytochrome c, which induces caspase activation [13]. Cellular oxidants are mainly by-products of endogenous processes: (1) mitochondrial ATP production, (2) phagocytosis, (3) *β*-oxidation of long-chain fatty acids (>C20), and (4) other metabolic pathways, such as inflammation [14, 15]. Normally, damages caused by free radicals are repaired by a class of molecules named antioxidants. However, when antioxidant defences are not adequate, that is, when excessive amounts of free radicals are generated, the cell undergoes oxidative stress. In this condition, several damages may occur at protein, enzyme, lipid, and nucleic acid levels. In the latter, the production of reactive singlet oxygen can react with all DNA bases. More in detail, when the single oxygen reacts with guanine, the process generates 8-oxo-7,8-dihydro-2′-deoxyguanosine (8-oxo-dG) [16]. While guanine normally pairs with cytosine, 8-oxo-dG pairs with adenine; thus, the resulting point mutation will be translated in a mutated protein.

Generally, cell damages may alter downstream cell signalling and cause a variety of diseases, such as cardiovascular diseases, neurodegenerative disorders, cancers, and also aging, including skin aging [17–25].

## 3. Environmental and Xenobiotic Agents Involved in Skin Aging

Aging is a complex biological process, as it induces progressive deterioration of anatomical structures and of the physiological functions of the organs [26]. The skin is the outermost barrier of the body and the biggest organ, and its changes are among the most visible signs of aging. Indeed, with aging, the skin loses some of its properties, such as elasticity, thickness, and colour [27]. The normal cellular oxidative metabolism can generate different by-products responsible for molecular damage, thus contributing to skin aging (intrinsic aging). However, it has been reported that up to 80–90% of skin aging is due to environmental and xenobiotic agents (extrinsic aging) [26, 28].

Several external factors may represent a cause of free radical production and consequently induce skin aging. Among them, it is worth mentioning air and water contaminants, tobacco smoke, different organic solvents, several drugs (such as bleomycin and gentamycin), kitchen scraps (i.e., used oil and fat), and heavy or transition metals (such as lead, cadmium, mercury, and iron) ([18] and references therein). In particular, air pollution includes biological and gaseous contaminants, as well as particulate. Pollution has been reported to exert deleterious effects on the skin in different ways [29]: (a) ultrafine particles can penetrate tissues and localize in mitochondria, thus inducing ROS generation [30]; (b) diesel exhaust particles induce activation of the inflammatory cascade in keratinocytes [31]; (c) pollutants are among the activators of the aryl hydrocarbon receptor (AhR), a cytosolic ligand-activated transcription factor that regulates cellular proliferation, inflammation, and melanogenesis [32]; and (d) pollutants can alter skin microflora [33, 34]. Another external stress factor is arsenic. This chemical element is widely present in food, water, air, and soil and is mostly found in its trivalent (As^3+^, such as sodium arsenite and arsenic trioxide) or pentavalent (As^5+^) inorganic form. There are many pieces of evidence demonstrating that the deleterious effects of trioxide arsenic are mostly due to its inorganic state, rather than to the organic form [35–37], since it induces the generation of free radicals in cells and, consequently, leads to oxidative stress, resulting in oxidative DNA damage and finally into apoptosis [38–42].

Tobacco smoking has been underestimated as stress factor for a long time and its damages have resulted more evident with the increase of life expectancy [43, 44]. Smoking dates back to as early as 5000 BC in the Americas in shamanistic rituals [45], but the first report that described a link between smoking and cancer was published in 1928 [46]. To date, in PubMed, there are more than 53,000 research entries on this theme. Despite that, millions of people continue to smoke and the risk of cancer, as well as premature skin aging, is ignored [47, 48].

Nowadays, sunlight is among the most harmful exogenous factors able to induce ROS formation. The spectrum of sunlight includes infrared energy (above 760 nm), visible light (400–760 nm), and ultraviolet (UV) light (below 400 nm) [49]. UV radiations can be further divided in UVA (400–315 nm), UVB (315–280 nm), and UVC (280–100 nm). Photobiological responses are mostly generated by exposure to UVB and UVA radiations. UV radiations are the major cause of stem cell DNA damage; they can contribute to depletion of stem cells and damage of stem cell niche, eventually leading to photoinduced skin aging [50]. In particular, in the UVB range, direct light absorption by DNA mainly results in dimerization reactions between adjacent pyrimidine bases. Nevertheless, UVA radiations are considered more dangerous than UVB as, although they are weakly absorbed by DNA, they can excite endogenous chromophores, leading to DNA damage. Moreover, several endogenous and exogenous molecules, once exposed to photoexcitation, can lead to ROS formation [51].

## 4. Skin Photoaging as a Consequence of Oxidative Stress

Exposure to UV irradiation induces photochronical generation of ROS that activates cell surface growth factors, cytokine receptors, and nicotinamide adenine dinucleotide phosphate (NADPH) oxidase [52]. This induces signal propagation within the cell through phosphorylation of tyrosine residues on the receptors and their associated adaptor proteins [53]. In particular, two nuclear transcription factors, activator protein 1 (AP-1) and nuclear factor-*κ*B (NF-*κ*B), involved in transcription of genes for matrix-degrading enzymes and proinflammatory cytokines, respectively, are activated [54]. These transcription factors are associated with skin dryness, pigmentation, laxity, deep wrinkling [55, 56], and apoptosis activation [57, 58].

However, photoaging is a cumulative process, as many factors contribute to it, such as the degree of sun exposure and skin pigment. Indeed, it has been demonstrated that people with lighter skin colour, living in sunny countries, developed more easily skin cancers, such as basal cell cancer, squamous cell carcinoma, and melanoma [54].

To hide the effects of aging, several people undergo plastic surgery. However, in most cases, this practice is expensive, very invasive, and could potentially lead to complications. In this context, development of new antiaging therapies is gaining more importance.

During the last decade, different studies demonstrated that chronological aging and photoaging activate the same oxidative stress-related pathways. However, photoaging accounts for about 80% of the aging-related adverse effects. In this context, antioxidants are known to inhibit damages induced by oxidation, caused by ROS, as described in this review in paragraph 5 [54, 59, 60].

## 5. Antioxidants as Antagonists of ROS in Skin Disorders

The aerobic world is characterized by high levels of toxic oxygen by-products. To survive in this adverse environment, the organism has evolved antioxidant systems to protect itself. Khlebnikov et al. defined the antioxidants as “any substance that directly scavenges ROS or indirectly acts to upregulate antioxidant defences or inhibit ROS production” [61]. However, antioxidants are also characterized by the ability to form a new, more stable radical, through intramolecular hydrogen bonding and further oxidation [62]. In addition, antioxidants can regulate gene expression inducing the translocation of the nuclear factor-erythroid 2-related factor 2 (Nrf-2) from the cytosol to the nucleus, upon dissociation from its inhibitor, Kelch-like erythroid cell-derived protein 1 (Keap-1). Once in the nucleus, Nrf-2, can bind antioxidant response elements (ARE) and induces the transcription of stress response genes, such as glutathione S-transferase (GST), heme oxygenase-1 (HO-1), and NAD(P)H: quinone acceptor oxidoreductase 1 (NQO1) [14, 63, 64]. The defence system of the cell from oxidative stress consists of an interacting network of different antioxidants that acts at different levels and with different mechanisms, which are summarized in Table 1.

**Figure 1:**
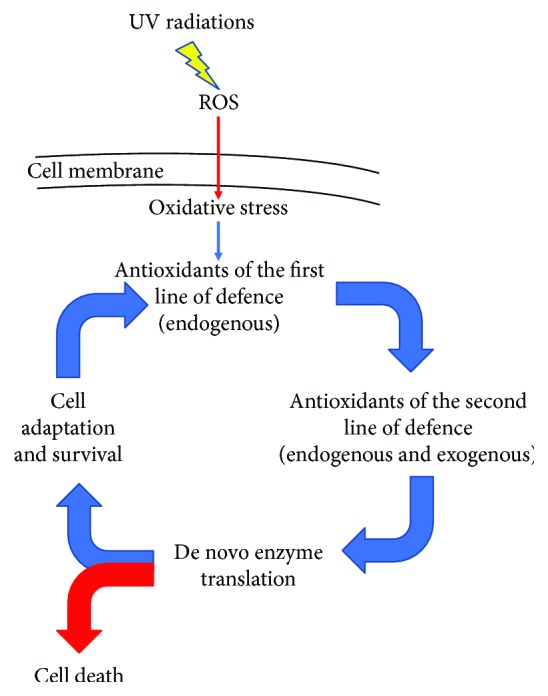
Schematic representation of the cell antioxidant response following oxidative stress injury. Upon UV radiations, ROS levels increase and oxidative stress is induced. Endogenous antioxidants suppress ROS formation and exogenous and endogenous antioxidants cooperate to suppress propagation reactions. Cell damages are repaired by de novo enzymes. Finally, if the cooperation among these antioxidant-related networks is able to counteract oxidative stress injury, the cell will survive after an adaptation process; otherwise, in case of prolonged or excessive stress, the cell will undergo cell death.

Antioxidants belonging to the first line of defence suppress the formation of free radicals, whereas those of the second line counteract the chain initiation and/or break the chain propagation reactions of free radicals. Following oxidative stress, the cell is able to induce the transcription and translation of de novo enzymes, involved in repair processes. If the cell is able to counteract the negative effects of stress injury, it will undergo adaptation and restore the physiological antioxidant levels. On the other hand, in case of prolonged or excessive stress, the cell will undergo programmed cell death, as schematically represented in Figure 1.

In general, antioxidants can be grouped as endogenous, that is, produced by the body, and exogenous, that is, obtained from the diet. The first class can be divided in enzymatic and nonenzymatic defences. The first group includes superoxide dismutase (SOD) [65], catalase [65], and glutathione peroxidase [65], whereas the nonenzymatic defenders include iron- and copper-binding extracellular proteins (e.g., albumin, transferrin, lactoferrin, haptoglobin, and ceruloplasmin) [66] as well as other cellular compounds (e.g., quinones, glutathione, uric acid, and bilirubin) [66].

Enzymatic and nonenzymatic defenders are complementary to each other, since they act against different oxidative species in different cellular compartments. Moreover, they may act in a synergistic way with the exogenous antioxidant systems. This last family of antioxidants can be divided into synthetic and natural antioxidants. Some examples for each class are reported in Table 2, with a focus on antioxidants able to protect the skin from photoaging.

## 6. Fruits and Vegetables as Powerful Sources of Antiaging Antioxidants

Exogenous supplementation with dietary antioxidants and/or skin pretreatment with antioxidant-based lotions before sun exposure might be a winning strategy against age-related skin oxidative damage [86]. Indeed, a regular intake of vitamins, polyunsaturated fatty acids, and polyphenols from plant sources has been shown to contribute to the prevention of age-related diseases. The search for effective natural compounds able to protect against the deleterious effects of photoaging has been intensified in recent years. Indeed, the list of molecules with antiaging potential extracted from different parts of a number of plant species is continuously growing [87, 88].

In this context, plants produce many secondary metabolites to protect themselves from UV radiations and these molecules can be used as natural antioxidants able to protect the skin from photoaging. These active compounds can protect the skin by (i) absorbing UV radiations, (ii) inhibiting free radical reactions induced by UV in cells, and (iii) modulating endogenous antioxidant and inflammatory systems [4, 89].

In the following part of the review, we will describe some of these natural antioxidants, the plants from which these compounds are normally extracted and their role in photoaging.

### 6.1. Phenolic Compounds

Natural compounds used against photoaging comprise phenolic compounds, including flavonoids (catechins, isoflavones, proanthocyanidins, and anthocyanins), phenolic acids (benzoic, gallic, and cinnamic acids), and stilbenes derived from plants such as tea, grape, bergamot, fernblock, rooibos, grapefruit, and red orange [4, 88, 90]. All these compounds can prevent penetration of radiations into the skin and, in addition, they can reduce inflammation, oxidative stress, and influence several signalling pathways in order to protect the skin against UV damage [4]. We recently demonstrated that two phenolic compounds, malvidin and cyanidin, extracted from fruits of the açai tree (*Euterpe oleracea* Mart.), a South American palm, were able to counteract UVA-induced oxidative stress in immortalized fibroblasts [74]. Indeed, the preincubation of UVA-irradiated BALB/3T3 cells with açai phenolic compounds interfered with ROS production and kept GSH levels and lipid peroxidation comparable to normal cellular levels [74]. In another paper, we showed the beneficial effects of water extracts from *Opuntia ficus-indica* L. cladodes on human keratinocytes [91]. In particular, the phenolic compounds eucomic and piscidic acids were found to be the main active molecules responsible for the protection of keratinocytes against the UVA-induced oxidative stress and apoptosis [91].

Several studies have demonstrated the health-promoting effect of grape (*Vitis vinifera*) against age-related diseases. This is due to the high content of phenolic compounds present in this plant. Indeed, grape seeds and peels constitute a rich source of polyphenols including quercetin, catechin, epicatechin, gallic acid, and oligomeric proanthocyanidins [4, 92]. Recently, it has been found that also grape extracts from the stems, a part of the grape tree rich in phenolic compounds, are able to reduce UVB-induced oxidative damage [93]. Indeed, the topical application of stem's grape extracts on mice skin before UVB treatment was able to prevent epidermal thickness, erythema, pigmentation, mast cell and inflammatory neutrophil infiltrations, collagen degradation, and the expression of COX-2, Nrf-2, and HO-1 genes [93].

Grape seeds are also rich in phytoalexin resveratrol (trans-3, 4′,5-trihydroxy-stilbene), a polyphenolic antioxidant with strong anti-inflammatory and antiproliferative activity [4, 87]. A single application of resveratrol on hairless mice's skin before exposure to UVB radiation led to the inhibition of skin edema, cyclooxygenase, and ornithine decarboxylase induction and lipid peroxidation in the skin [4, 76]. Interestingly, the human skin has been shown to have specific binding sites for resveratrol. As recently reviewed by Davinelli and colleagues [94], the interaction of resveratrol with the specific binding partner is able to block apoptotic events and mitochondrial dysfunctions in keratinocytes, delaying skin aging. In human keratinocytes, resveratrol can also modulate cytokine (IL-6, IL-8, and TNF-*α*) levels and stimulate the expression of HSP70, a factor important for cell repair and also for cytoprotection ([75] and references therein). However, one should keep in mind that resveratrol has very low solubility and high sensitivity to oxidation, thus making this molecule very unstable ([94] and references therein).

Human intervention studies have also been carried out, and most of them have focused on the supplementation of epigallocatechin gallate (EGCG) from green tea. In one of these studies, human subjects received 800 mg of EGCG, in one dose or divided in two doses, and this treatment consistently reduced the erythema size caused by the exposure to UV radiation [95]. Attention should be paid to the local EGCG concentration as, when tested in nM concentrations, it acts as antioxidant, whereas when tested in the *μ*M concentration range, EGCG acts as a prooxidant [96]. In another study on human subjects, high dose of flavanols from cocoa powder alleviated the erythema size upon UV radiation exposure [97].

### 6.2. Vitamin C

Vitamin C (ascorbic acid) is an essential cofactor in several enzymatic reactions but, since it cannot be synthesized by the human body, it has to be introduced in the organism by diet [98]. The antioxidant activity of ascorbic acid, which is found in fruits such as acerola, orange, lemon, tangerine, and tomato, makes it a good candidate as a protective compound against UV irradiation [99, 100]. Tomato (*Solanum lycopersicum*) fruits are a good source of ascorbic acid. Notably, we recently demonstrated the ability of an ascorbic acid-enriched tomato genotype to fight the oxidative stress induced by UVA in normal human keratinocytes [83]. In particular, pretreatment of cells with ascorbic acid or with tomato extracts before UVA exposure was able to maintain ROS, GSH, and lipid peroxidation levels at the basal levels and there was no evidence of apoptosis or inflammation [83]. These findings have been corroborated by Pullar et al., who demonstrated that ascorbic acid prevents lipid peroxidation and protects keratinocyte exposed to UV radiation from apoptosis [100]. In humans, it has been found that ascorbic acid acts as a photoprotectant at doses above the minimal erythema dose and that stimulates collagen synthesis, protects against damage from UVA/B radiation, and mitigates inflammation in the skin [86, 99, 100]. Finally, it has been demonstrated that topical application of an antioxidant mixture containing grape seed extract, vitamin E, ubiquinone, and ascorbic acid was able to protect the human skin against infrared A radiation-induced MMP-1 upregulation [101]. Unfortunately, many factors influence vitamin C stability, such as its concentration, temperature, and the pH used for aqueous formulations (which should be used at a pH lower than its pKa) [102].

### 6.3. Carotenoids

Carotenoids are dietary antioxidants that have demonstrated photoprotective activity. In plants, these compounds are components of the photosynthetic machinery where they act as accessory light-harvesting pigments and protect from photooxidative damage [103]. The photoprotective effects of several carotenoids have been investigated by intervention studies in humans, in which a carotenoid-rich diet has been investigated for its ability to decrease the erythema size upon UV radiation exposure [104–106], even though a long period (at least ten weeks) is necessary for successful intervention [106].

Among carotenoids, *β*-carotene, lycopene, canthaxanthin, and lutein that are derived from different plant sources such as tomato, carrots, and algae are the most abundant [107]. Carotenoids capsanthin and capsorubin from red pepper (*Capsicum annuum* L.) fruits have also been found to have protective properties against UVB-induced DNA damage in human dermal fibroblasts [88, 108]. Indeed, cell pretreatment with these carotenoids decreased the formation of UVB-induced DNA strand breaks and counteracted caspase-3 activation [108]. Studies on *β*-carotene protective effects go back to early ‘70s as this molecule showed very promising results. In 1996, a trial was reported on 12 years of supplementation with *β*-carotene (50 mg on alternate days) on healthy men but did not show any protective effect from melanoma insurgence [109].

Moreover, safety concerns have been recently raised with regard to its supplementation for over long periods of time [79]. Although the photoprotective effects of beta-carotene are thought to originate from its antioxidant properties, some studies documented prooxidant effects of *β*-carotene. For this reason, recent studies are focused on carotenoids other than *β*-carotene, such as lycopene, the primary carotenoid in tomatoes. We recently demonstrated that tomato extracts, rich in lycopene, are effective in counteracting the detrimental effects induced by oxidative stress caused by treatments with sodium arsenite on different human cell lines [80]. In particular, we found that carotenoids extracted from both fresh and processed tomato fruits showed cytoprotective activity, were able to mitigate ROS production induced by oxidative stress, and prevented GSH depletion and lipid peroxidation [80].

It has been demonstrated that lycopene protects against various skin alterations induced by UV radiation [90]. As an example, lycopene has been shown to have a role in the prevention of skin cancer. Indeed, lycopene preexposure on UVB-irradiated human keratinocytes was found to play a diversified role in UVB-irradiated keratinocytes, depending on the level of damage, correcting the injury in mild photodamaged cells and acting as a cytotoxic agent in preneoplastic cells [110]. In particular, in irradiated keratinocytes, lycopene pretreatment resulted in the overexpression of BAX gene, a cell cycle delay at S-phase transition, and in a consequent decrease of cells in G0/G1 phase [110].

In a placebo-controlled, double-blinded, randomized study, oral supplementation with lycopene-rich tomato nutrient complex (TNC) and lutein has been shown to be able to protect the human skin against UVA and UVA/UVB radiations [79]. In particular, oral supplementation with TNC inhibited UV-induced upregulation of the genes heme oxygenase-1, intercellular adhesion molecule 1, and matrix metallopeptidase 1, indicators of oxidative stress, photodermatoses and photoaging [79].

Several studies suggested that the protection from UV radiation is more effective upon treatment with combined tomato antioxidant compounds, compared to the effects of lycopene treatment alone, and this is probably due to a synergistic effect of the different tomato phytonutrients [79, 111]. This could be related to the fact that the interaction between structurally different molecules, endowed with different antioxidant properties, may provide a more comprehensive protection against oxidative injury [84].

Accordingly, consumption of tomato paste has been shown, in a randomized controlled study, to protect against acute and long-term photodamage [112]. In particular, supplementation of tomato paste before UV exposure was able to dampen skin erythema and to reduce mitochondrial DNA damage [112, 113]. Moreover, it has been recently showed that, in hairless and immunocompetent mice, tomato consumption was able to protect against the development of UVB-induced keratinocyte carcinoma [113].

## 7. Conclusions

Skin photoaging is a consequence of the oxidative stress generated upon exposure to UV radiation. However, the skin is normally protected from the negative effects of oxidative stress by endogenous antioxidant systems, which, unfortunately, undergo a progressive decline during aging. Several lines of evidence support the hypothesis that secondary metabolites from plants act as natural antioxidants able to decrease or retard the development and progression of life style-related diseases.

**Figure 2:**
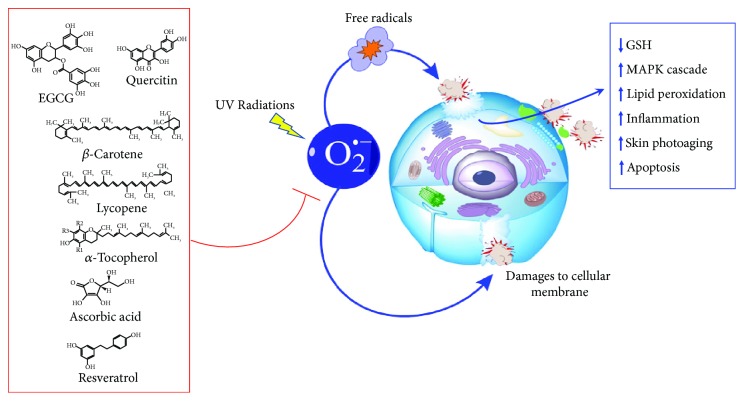
Cartoon representing cellular responses to oxidative stress in the presence (red lines) or in the absence (blue lines) of antioxidants. After oxidative stress induction by UV radiations, there is an increase in free radicals, which, in turn, induces different responses in the cell, such as depletion in GSH, activation of MAPK cascade, increase in lipid peroxidation, inflammation, skin photoaging, and apoptosis. All these processes can be inhibited or counteracted by antioxidant's activity (reported in the red box).

The intake of dietary antioxidants plays a fundamental role in the protection against oxidative injury; therefore, a correct diet is crucial to extend lifespan. Accordingly, several *in vitro*, *in vivo*, and human intervention studies demonstrated that antioxidants deriving from natural products, most of them assumed with the Mediterranean diet, are particularly effective in the protection of skin from photoaging, as schematically reported in Figure 2.

However, the use of natural antioxidants not only is restricted to oral diet but also includes a potential topical use against UV radiations. This is possible as some natural compounds show UV absorption properties and act as antioxidants, thus reducing the damaging effects of UV radiation exposure. Thus, increasing the antioxidant capacity of skin cells by using exogenous antioxidants could be a valuable strategy for preventing UV-induced skin damage.

## Figures and Tables

**Table 1:** Defence levels and mechanism of action of antioxidants.

The first line of defence		The second line of defence		The third line of defence	
Antioxidant	Mechanism of action	Antioxidant	Mechanism of action	De novo enzymes	Mechanism of action
Superoxide dismutase	O_2_^•−^ → H_2_O_2_	Ascorbic acid	Chain breaking: donate an electron to the free radical	Polymerases	DNA repair
Catalase	2H_2_O_2_ → O_2_ + H_2_O	Uric acid		Glycosylases	
Glutathione peroxidase	H_2_O_2_ + GSH → GSSG + H_2_O	Glutathione		Nucleases	
Transferrin	Metal chelators or sequesters	α-Tocopherol			
Caeruloplasmin		Ubiquinol		Proteinases	Protein proteolysis
		*β*-Carotene	Incorporation of free radical	Proteases	
		Lycopene		Peptidases	

**Table 2:** Antioxidants involved in protection from photoaging.

Antioxidants	Class	Bioactive compound	Skin protection from photoaging	Ref
Synthetic	Nitroxides (mimetics of SOD)	Tempol	Protection from UVA- and UVB-induced damage *in vitro* and *in vivo* Inhibition of extracellular matrix degradation and preservation of collagen production *in vitro*	[67–70]
	Coenzyme Q analogues	Idebenone	Protection from oxidative stress damage in living skin Suppression of sunburn cell formation	[71]

Natural	Flavonoids	Quercetin	Inhibition of UV-induced inflammation in primary human keratinocytes Protection of mice skin from UV radiation-induced damage	[72, 73]
		Malvidin and Cyanidin derivatives	Protection of murine fibroblast from UVA damages	[74]
	Polyphenols	Resveratrol	Protection of HaCaT cells from UVB irradiation through attenuation of the caspase pathway Counteraction of UVB damages in hairless mice Reduction of skin wrinkling and skin oxidative stress	[75–77]
	Carotenoids	*β*-Carotene	Prevention and repair from photoaging Protection of human skin against UV radiation in human clinical studies	[78–80]
		Lycopene		
		Luthein		
	Vitamins	Vitamin C	Protection of HaCaT cells from UVA irradiation through attenuation of inflammation and activation of apoptosis Antioxidant, photoprotection, antiaging, antipigmentary effects on the skin	[81–84]
		Vitamin E	Skin photoprotection against UV-induced oxidative stress	[84, 85]
